# Efficacy of a Topical Formulation on Skin Hydration and Barrier Function in Individuals With Xerosis: A 28‐Day Randomized, Split‐Leg, Untreated‐Controlled Trial

**DOI:** 10.1111/jocd.70898

**Published:** 2026-05-10

**Authors:** Daniel Morgado‐Carrasco, Pablo Balado‐Simó, Fernando Vivancos Cuadras, Marta Furmanczyk, Jesús Delgado Ojeda

**Affiliations:** ^1^ Dermatology Department Hospital Clínic de Barcelona, Universitat de Barcelona Barcelona Spain; ^2^ Dermatology Department Hospital de Figueres, Fundació Salut Empordà Figueres Spain; ^3^ Lacer S.A. Barcelona Spain

**Keywords:** dipotassium glycyrrhizate, emollients, niacinamide, panthenol, skin barrier function, xerosis cutis

## Abstract

**Background:**

Xerosis cutis is characterized by impaired epidermal barrier function, increased transepidermal water loss (TEWL), reduced hydration, and symptoms such as pruritus.

**Aims:**

To evaluate the efficacy, tolerability, and subjective perception of a topical formulation containing panthenol, niacinamide, and dipotassium glycyrrhizate in improving skin hydration and barrier function in subjects with dry, sensitive skin.

**Methods:**

In this randomized, intra‐individual, split‐leg clinical trial, subjects with xerosis applied the test emollient twice daily for 28 days to one pretibial area. The contralateral leg remained untreated as control. Assessments were performed at baseline (T0), 30 min, 24 h, 48 h, day 7 and day 28. Primary outcomes were corneometric stratum corneum hydration and TEWL. Secondary outcomes included deep skin conductance (“deep moisturization”), desquamation index, surface microrelief regularity (SEr), scaliness (SEsc), dermatologist‐rated clinical signs, and participant self‐assessment.

**Results:**

Thirty‐five participants (31 women; mean age 47.3 ± 2.0 years) were included. Compared with baseline (hydration: 21.3 ± 1.0 c.u., 95% CI: 19.3–23.3; TEWL: 9.2 ± 0.3 g/h/m^2^, 95% CI: 8.6–9.8) and untreated control, the treated leg showed greater corneometric hydration at all time points: 66.2% (33.9 ± 1.4 c.u., 95% CI: 31.1–36.7) at day 7 and 74.1% (35.2 ± 1.2 c.u., 95% CI: 32.8–37.6) at day 28 (all *p* < 0.001). TEWL decreased by 11.3% (8.2 ± 0.2 g/h/m^2^, 95% CI: 7.8–8.6) and 13.5% (7.9 ± 0.2 g/h/m^2^, 95% CI: 7.5–8.3), respectively (all *p* < 0.05). Deep moisturization increased by 5.7% (39.5 ± 0.9 μS at day 7, 95% CI: 37.7–41.3) and 7.3% (41.1 ± 0.8 μS at day 28, 95% CI: 39.5–42.7) (*p* < 0.05). The desquamation index decreased by 13.0% (29.50% ± 1.67% at day 7, 95% CI: 26.1–32.9) and 15.8% (26.77% ± 1.25% at day 28, 95% CI: 24.2–29.3), SEr increased by 46.9% (3.38 ± 0.15 a.u. at day 7, 95% CI: 3.08–3.68) and 54.2% (3.47 ± 0.15 a.u. at day 28, 95% CI: 3.17–3.77), and SEsc decreased by 67.0% (0.81 ± 0.12 a.u. at day 7, 95% CI: 0.57–1.05) and 76.3% (0.51 ± 0.07 a.u. at day 28, 95% CI: 0.37–0.65), respectively (all *p* < 0.05). Dermatologist assessments at all time points showed significant clinical improvement in dryness and pruritus in all participants (*p* < 0.001). Complete resolution was observed in all participants according to the 5‐point scale, though this may reflect a floor effect given mild baseline scores.

**Conclusions:**

The topical formulation appeared to be an effective and well‐tolerated emollient; however, the absence of a vehicle control precludes attribution of effects specifically to the active ingredients. Patient‐reported outcomes should be interpreted with caution due to the unblinded design.

## 1 Introduction

The skin functions as a complex biological barrier. The stratum corneum (SC) exhibits a distinctive “bricks and mortar” structure, with protein‐rich corneocytes embedded within a matrix of intercellular lipids [1, 2, 3]. This architecture is crucial for minimizing transepidermal water loss (TEWL) and maintaining hydration within the viable epidermis.

Xerosis cutis, a prevalent dermatological condition, arises from impairment of the epidermal barrier and clinically manifests as scaling, roughness, and pruritus [4]. Its pathophysiology involves a deficiency in essential stratum corneum lipids such as ceramides, cholesterol, and free fatty acids, together with reduced levels of natural moisturizing factors (NMFs) and increased TEWL [5, 6]. These biophysical alterations translate into substantial patient discomfort and may exacerbate or contribute to chronic inflammatory dermatoses [3, 7, 8]. While conventional management typically relies on basic emollients and cutaneous hydration [9, 10], there is a growing need for multi‐active formulations that facilitate functional barrier enhancement and address associated symptoms such as pruritus. Panthenol (provitamin B5) exhibits humectant, anti‐inflammatory, and epithelial restorative properties [11, 12], while niacinamide (vitamin B3) has been shown to stimulate ceramide biosynthesis, enhance barrier lipid production, and reduce inflammatory cytokine release [13, 14]. In addition, dipotassium glycyrrhizate (DPG) has demonstrated anti‐inflammatory and soothing effects via inhibition of NF‐κB activation and downregulation of interleukins implicated in pruritus and irritation [15, 16]. A topical formulation combining these three agents could differentiate itself from standard‐of‐care moisturizers (urea‐based and ceramide‐containing creams, among others) by offering a multi‐effect approach.

This study aimed to assess the clinical efficacy, instrumental outcomes, and user perception of a novel topical formulation containing panthenol, niacinamide, and DPG in individuals with clinically confirmed xerosis.

## 2 Materials and Methods

### 2.1 Study Design and Participants

This single‐center, randomized, intra‐subject controlled clinical trial was conducted in accordance with the ethical principles of the Declaration of Helsinki. The study was registered on ClinicalTrials.gov (NCT07393191). The protocol was approved by *Comitato Etico Indipendente per le Indagini Cliniche Non Farmacologiche* on February 5th, 2025 (reference number 2025/03). All participants provided written informed consent before the commencement of any study‐related procedures.

Male and female patients of Caucasian ethnicity, aged 18–65 years were eligible. Inclusion criteria required clinically diagnosed dry (corneometry value < 30 corneometric units [c.u.]) or very dry skin (< 25 c.u.), with sensitive and cracked skin on the legs, accompanied by clinical signs and symptoms including cutaneous dryness, desquamation, tightness and pruritus. Exclusion criteria included acute or chronic diseases that could interfere with study outcomes, concurrent participation in other clinical trials, known allergy to any of the product ingredients, and pregnancy or breastfeeding.

The sample size was set at 35 subjects to ensure that a minimum of 30 participants would complete the 28‐day study period (allowing for a 14% dropout rate). This target provides 80% power to detect a mean change in hydration of 5.0 c.u. with a standard deviation of 9.0 c.u. (Cohen's), based on standard effect sizes observed in intra‐subject controlled trials for xerosis management.

### 2.2 Intervention

The product under investigation was a cosmetic formulation (Talquistina Regenera+, Lacer, Italfarmaco, Italy) containing panthenol, niacinamide, and dipotassium glycyrrhizate. Randomization was performed using a predefined computer‐generated list to assign treatment to the right or left leg. Participants were then instructed to apply one gram (gr) of the product twice daily to a 20 × 10 cm area on the pretibial region of one leg for 28 consecutive days. The contralateral leg remained untreated and served as the intra‐subject control, allowing for direct comparison under identical systemic and environmental conditions. Participant compliance was monitored by weighing the product containers at baseline and at the end of the trial. They were verbally interviewed at each follow‐up visit to reinforce adherence.

Participants were provided with a standardized neutral detergent and instructed to use it exclusively on both legs.

### 2.3 Efficacy and Safety Assessments

All instrumental evaluations were conducted in a controlled environment (temperature 22°C ± 2°C; relative humidity 50% ± 10%) after a 15‐min acclimatization period. Assessments were performed at baseline (T0) and after 30 min (T30min), 24 h (T24h), 48 h (T48h), 7 days (T7), and 28 days (T28) of product use.

The primary endpoints were stratum corneum hydration and epidermal barrier integrity. Surface hydration was measured using a Corneometer CM 825 (Courage+Khazaka, Germany) at T0, T7, and T28. Transepidermal water loss (TEWL) was measured to evaluate barrier function using a Tewameter TM 300 (Courage+Khazaka, Germany). TEWL was assessed at all scheduled time points on both treated and untreated legs.

Secondary endpoints included deeper epidermal hydration, surface morphology, clinical evaluation, and patient‐reported outcomes. Deep skin hydration was quantified using conductance analysis with a DermaLab‐Pin probe (CORTEX TECHNOLOGY, Denmark), which uses pin‐array electrodes to assess water content in the lower epidermis. The device gives information about the hydration state by measuring the skin conductance. The measuring probe contains an array of eight pins and is designed to facilitate hydration measurements and to minimize water accumulation under the electrodes. This parameter is a validated proxy for local tissue water content at a depth of 500 μm, providing an assessment of the viable epidermis's hydration state, which is distinct from the superficial hydration of the stratum corneum [17]. Conductance measurements may be influenced by total fluid volume, not exclusively intracellular hydration.

To evaluate changes in desquamation and skin surface topography, Corneofix F20 adhesive foils were applied to collect corneocytes, which were then imaged using a Visioscan VC 20plus camera. Quantitative analysis of these images was performed using SELS (Surface Evaluation of the Living Skin) software, which computed the desquamation index as well as scaliness (SEsc) and surface microrelief regularity (SEr) (historically termed ‘Surface Roughness’) parameters. It is important to note that within the SELS algorithm, the SEr parameter indicates the presence of a well‐structured skin surface; thus, higher values reflect a more regular and homogeneous microrelief rather than clinical roughness.

In addition to instrumental assessments, a board‐certified dermatologist also performed blinded clinical evaluations, rating both physical signs (dryness, desquamation) and functional symptoms (tightness, pruritus) using a standardized 5‐point ordinal scale, where 0 indicated absence and 4 represented severe involvement (0 = none, 1 = very mild, 2 = mild, 3 = moderate, 4 = severe). Participants completed structured questionnaires at multiple time points (T0, T24h, T48h, T7, and T28) to record their subjective perceptions of efficacy and cosmetic acceptability.

Safety and tolerability were monitored throughout the study. The investigator recorded any adverse events (AEs) at follow‐up appointments (T0, T24h, T48h, T7, and T28) and assessed their causal relationship to the investigational product. Tolerability was further evaluated through participant feedback collected via self‐assessment questionnaires at the end of the study period.

### 2.4 Statistical Analysis

Efficacy analysis was performed on the per‐protocol population, defined as all subjects who completed the study without major protocol violations. Safety analysis was based on the intent‐to‐treat population, including all subjects who received at least one product application. Results are presented as values ± standard error (SE), unless otherwise specified. The choice between parametric and non‐parametric tests was determined by the data type and distribution. Continuous instrumental data (hydration, TEWL, conductance) were first assessed for normality using the Shapiro–Wilk test; as they met normality assumptions, parametric tests (repeated‐measures ANOVA with Tukey–Kramer post hoc) were applied. Non‐parametric tests (Friedman test and Wilcoxon signed‐rank test) were used for clinical sign scores (0–4 scale) to respect their ordinal nature and lack of normal distribution. Intragroup (post‐treatment vs. baseline) and intergroup (treated vs. untreated site) comparisons were performed. A *p* < 0.05 was considered statistically significant. All statistical analyses were conducted using NCSS 10 software.

## 3 Results

**FIGURE 1:**
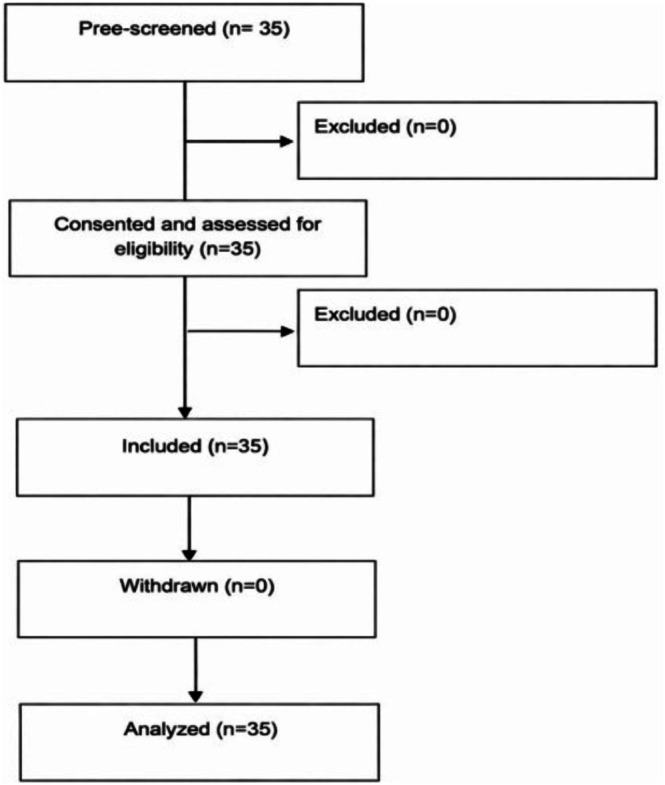
Trial flowchart.

Thirty‐five participants took part (Figure 1); 31 (89%) were female. The mean (± SD) age was 47.3 ± 2.3 years (range, 23–65 years), and all participants completed the 28‐day study period without any major protocol violations or dropouts. At baseline, there were no statistically significant differences between the leg sites designated for treatment and control across all instrumental and clinical parameters (Table 1).

**TABLE 1:** Baseline demographics and clinical characteristics of the study population (*N* = 35).

Characteristic	Value
Age, years (Mean ± SD)	47.3 ± 13.8
Sex, *n* (%)	
Female	31 (89)
Male	4 (11)
Ethnicity, *n* (%)	
Caucasian	35 (100)
Baseline skin hydration (c.u.), Mean ± SE	
Treated site	21.3 ± 1.0
Untreated site	21.1 ± 1.0
Baseline TEWL (g/h/m2), Mean ± SE	
Treated site	9.2 ± 0.3
Untreated site	9.2 ± 0.3
Baseline skin scaliness (SEsc, a.u.), Mean ± SE	
Treated site	3.10 ± 0.34
Untreated site	3.16 ± 0.31

Abbreviations: a.u., arbitrary units; c.u., corneometric units; SD, standard deviation; SE, standard error; TEWL, transepidermal water loss.

### 3.1 Primary Outcomes

#### 3.1.1 Stratum Corneum Hydration

**FIGURE 2:**
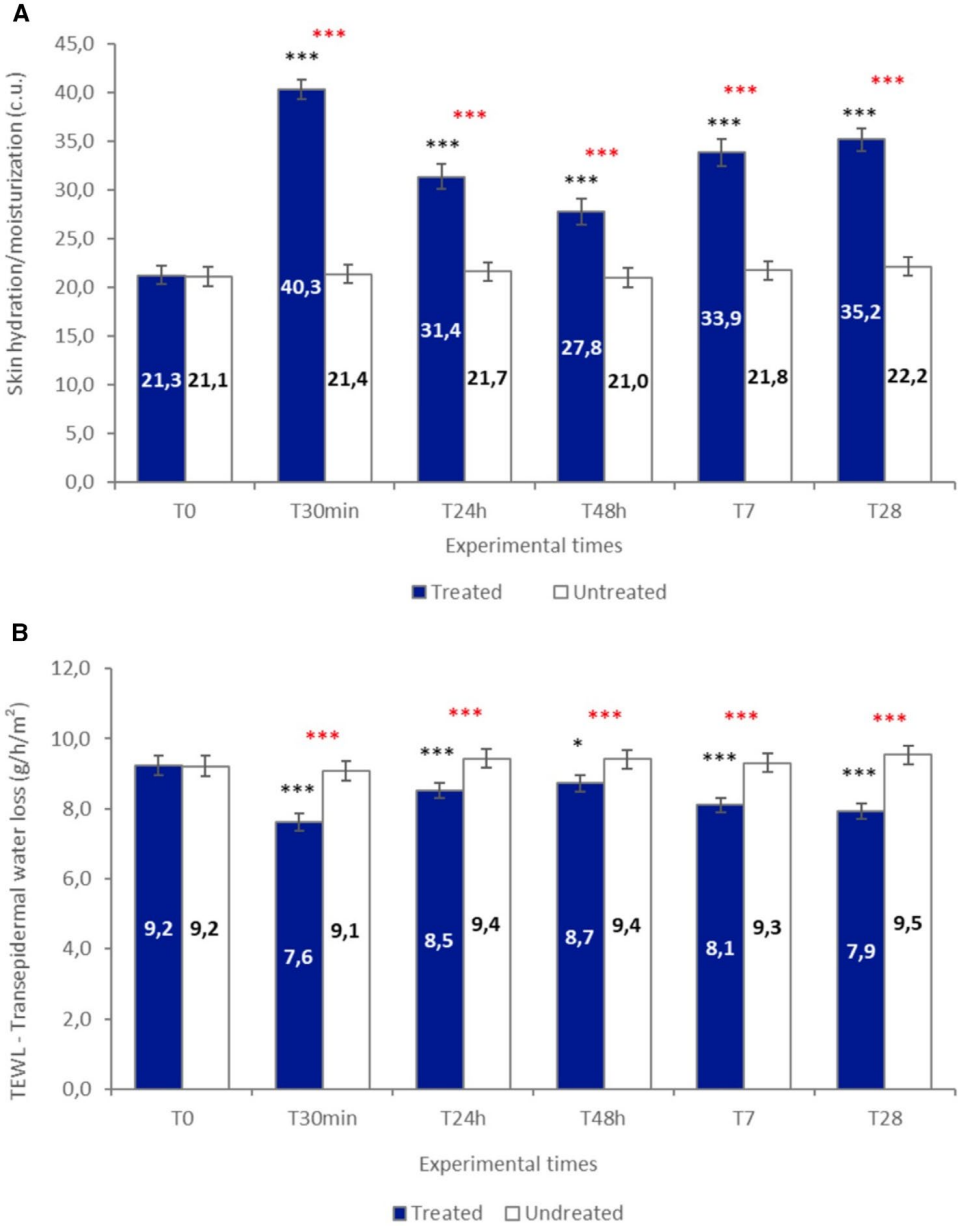
Skin hydration and transepidermal water loss. (A) As measured by corneometry, baseline hydration levels on the treated leg (21.3 ± 1.0 c.u., 95% CI: 19.3–23.3) increased by 102.1% (40.3 ± 1.0 c.u., 95% CI: 38.3–42.3) at T30min, 51.4% (31.4 ± 1.3 c.u., 95% CI: 28.8–34.0) at 24 h, 32.0% (27.8 ± 1.3 c.u., 95% CI: 25.2–30.4) at 48 h, 66.2% (33.9 ± 1.4 c.u., 95% CI: 31.1–36.7) at T7, and 74.1% (35.2 ± 1.2 c.u., 95% CI: 32.8–37.6) at T28 (*p* < 0.001). No significant variations were seen in the untreated (control) area. Data are expressed as mean ± SE. (B) A significant decrease of transepidermal water loss (TEWL) was reported at each experimental monitored check, both compared to baseline (T0) and to the untreated (control) area. Baseline TEWL (9.2 ± 0.3 g/h/m^2^, 95% CI: 8.6–9.8) decreased by 16.9% (7.6 ± 0.3 g/h/m^2^, 95% CI: 7.0–8.2) at T30min, 6.9% (8.6 ± 0.3 g/h/m^2^, 95% CI: 8.0–9.2) at T24h, 5.4% (8.7 ± 0.3 g/h/m^2^, 95% CI: 8.1–9.3) at T48h, 12.0% (8.1 ± 0.2 g/h/m^2^, 95% CI: 7.7–8.5) at T7, and 14.1% (7.9 ± 0.2 g/h/m^2^, 95% CI: 7.5–8.3) at T28 (all *p* < 0.001). No significant variations are monitored in the untreated (control) area. Data are expressed as mean ± SE. The intra‐group (**black** asterisks) and inter‐group (red asterisks) statistical analysis is reported as follows: *p < 0.05; **p < 0.01; ***p < 0.001.

Topical application of the test formulation led to a statistically significant improvement in stratum corneum hydration across all post‐treatment time points. As measured by corneometry, baseline hydration levels on the treated leg (21.3 ± 1.0 c.u.; 95% CI: 19.3–23.3) increased by 102.1% (40.3 ± 1.0 c.u.; 95% CI: 38.3–42.3) at T30min, 51.4% (31.4 ± 1.3 c.u.; 95% CI: 28.8–34.0) at 24 h, 32.0% (27.8 ± 1.3 c.u.; 95% CI: 25.2–30.4) at 48 h, 66.2% (33.9 ± 1.4 c.u.; 95% CI: 31.1–36.7) at T7, and 74.1% (35.2 ± 1.2 c.u.; 95% CI: 32.8–37.6) at T28 (all *p* < 0.001) (Figure 2 and Table 2). No meaningful changes in hydration were observed at any time point on the untreated leg.

**TABLE 2:** Summary of key instrumental and clinical efficacy outcomes.

Parameter	Group	Baseline [95% CI]	Day 7 [95% CI]	Day 28 [95% CI]	*p* (T0 vs. T28)	*p* (Tr vs. Unt at T28)
Skin hydration (c.u.)	Treated	21.3 ± 1.0 [19.3, 23.3]	33.9 ± 1.4 [31.1, 36.7]	35.2 ± 1.2 [32.8, 37.6]	*p* < 0.001	*p* < 0.001
	Untreated	21.1 ± 1.0 [19.1, 23.1]	21.8 ± 1.0 [19.8, 23.8]	22.2 ± 0.9 [20.4, 24.0]	*p* = 0.146	
TEWL (g/h/m2)	Treated	9.2 ± 0.3 [8.6, 9.8]	8.1 ± 0.2 [7.7, 8.5]	7.9 ± 0.2 [7.5, 8.3]	*p* < 0.001	*p* < 0.001
	Untreated	9.2 ± 0.3 [8.6, 9.8]	9.3 ± 0.3 [8.7, 9.9]	9.5 ± 0.3 [8.9, 10.1]	*p* = 0.111	
Deep moisturization (μS)	Treated	33.8 ± 0.8 [32.2, 35.4]	39.5 ± 0.9 [37.7, 41.3]	41.1 ± 0.8 [39.5, 42.7]	*p* < 0.001	*p* < 0.001
	Untreated	33.8 ± 0.8 [32.2, 35.4]	33.7 ± 0.8 [32.1, 35.3]	34.5 ± 0.8 [32.9, 36.1]	*p* = 0.356	
Desquamation index (%)	Treated	42.54 ± 1.35 [39.80, 45.28]	29.50 ± 1.67 [26.11, 32.89]	26.77 ± 1.25 [24.23, 29.31]	*p* < 0.001	*p* < 0.001
	Untreated	41.85 ± 1.43 [38.94, 44.76]	40.95 ± 1.48 [37.94, 43.96]	43.51 ± 1.55 [40.36, 46.66]	*p* = 0.420	
Skin surface microrelief regularity (SEr)*	Treated	2.36 ± 0.09 [2.18, 2.54]	3.38 ± 0.15 [3.08, 3.68]	3.47 ± 0.15 [3.17, 3.77]	*p* < 0.001	*p* < 0.001
	Untreated	2.48 ± 0.11 [2.26, 2.70]	2.52 ± 0.12 [2.28, 2.76]	2.59 ± 0.10 [2.39, 2.79]	*p* = 0.677	
Skin scaliness	Treated	3.10 ± 0.34 [2.41, 3.79]	0.81 ± 0.12 [0.57, 1.05]	0.51 ± 0.07 [0.37, 0.65]	*p* < 0.001	*p* < 0.001
	Untreated	3.16 ± 0.31 [2.53, 3.79]	2.90 ± 0.31 [2.27, 3.53]	2.68 ± 0.22 [2.23, 3.13]	*p* = 0.080	
Clinical dryness (0–4)	Treated	2.1 ± 0.1 [1.9, 2.3]	0.2 ± 0.1 [0.0, 0.4]	0.0 ± 0.0 [0.0, 0.0]	*p* < 0.001	*p* < 0.001
	Untreated	2.1 ± 0.1 [1.9, 2.3]	2.1 ± 0.1 [1.9, 2.3]	2.2 ± 0.1 [2.0, 2.4]	*p* = 0.240	
Clinical desquamation (0–4)	Treated	1.5 ± 0.1 [1.3, 1.7]	0.1 ± 0.1 [0.0, 0.3]	0.0 ± 0.0 [0.0, 0.0]	*p* < 0.001	*p* < 0.001
	Untreated	1.5 ± 0.1 [1.3, 1.7]	1.5 ± 0.1 [1.3, 1.7]	1.6 ± 0.1 [1.4, 1.8]	*p* = 0.063	

Abbreviations: T0, baseline; T28, Day 28; T7, Day 7; Tr, treated; Unt, untreated.

* Historically termed ‘Surface Roughness’.

#### 3.1.2 Transepidermal Water Loss

A significant reduction in TEWL was recorded on the treated leg, indicating improved barrier function. Baseline TEWL (9.2 ± 0.3 g/h/m^2^; 95% CI: 8.6–9.8) decreased by 16.9% (7.6 ± 0.3 g/h/m^2^; 95% CI: 7.0–8.2) at T30min, 6.9% (8.6 ± 0.3 g/h/m^2^; 95% CI: 8.0–9.2) at T24h, 5.4% (8.7 ± 0.3 g/h/m^2^; 95% CI: 8.1–9.3) at T48h, 12.0% (8.1 ± 0.2 g/h/m^2^; 95% CI: 7.7–8.5) at T7, and 14.1% (7.9 ± 0.2 g/h/m^2^; 95% CI: 7.5–8.3) at T28 (all *p* < 0.001) (Figure 2). TEWL values on the untreated side showed a slight increase over time, from 9.2 ± 0.3 g/h/m^2^ (95% CI: 8.6–9.8) at baseline to 9.5 ± 0.3 g/h/m^2^ (95% CI: 8.9–10.1) at day 28, although this variation was small in absolute magnitude (*p* = 0.111).

### 3.2 Secondary Outcomes

#### 3.2.1 Deeper Epidermal Hydration

Deeper epidermal hydration, as assessed by conductance measurements, showed progressive improvement. On the treated leg, baseline values (33.8 ± 0.8 μS; 95% CI: 32.2–35.4) increased by 16.9% (39.5 ± 0.9 μS; 95% CI: 37.7–41.3) at T7 and 21.6% (41.1 ± 0.8 μS; 95% CI: 39.5–42.7) at T28, both statistically significant in comparison to baseline and to the untreated control (*p* < 0.05).

#### 3.2.2 Skin Desquamation, Roughness and Scaliness

Skin desquamation, evaluated via image‐based analysis of corneocyte collection, showed marked improvement on the treated area. The baseline desquamation index (42.54% ± 1.35%; 95% CI: 39.80–45.28) decreased to 29.50% ± 1.67% at T7 (95% CI: 26.11–32.89) and to 26.77% ± 1.25% at T28 (95% CI: 24.23–29.31) (*p* < 0.001 for both), with no notable variation on the untreated leg.

In the SELS system, higher SEr values correspond to a more uniform and regular skin surface pattern. Baseline SEr (2.36 ± 0.09; 95% CI: 2.18–2.54) increased to 3.38 ± 0.15 at T7 (95% CI: 3.08–3.68) and to 3.47 ± 0.15 at T28 (95% CI: 3.17–3.77) (*p* < 0.001), consistent with improved surface regularity. Simultaneously, baseline scaliness (SEsc) values (3.10 ± 0.34; 95% CI: 2.41–3.79) declined significantly to 0.81 ± 0.12 at T7 (95% CI: 0.57–1.05) and to 0.51 ± 0.07 at T28 (95% CI: 0.37–0.65) (*p* < 0.001), supporting a reduction in visible desquamation. These differences were statistically significant when compared with the untreated site (*p* < 0.001 at both time points).

#### 3.2.3 Clinical Assessments

Clinical assessment showed a statistically significant reduction in all xerotic signs and symptoms compared to both baseline and the untreated control area (*p* < 0.001, Tables 2 and S2). At baseline (T0), participants presented with mild‐to‐moderate clinical dryness (mean score 2.1 ± 0.1 on a 0–4 scale; 95% CI: 1.9–2.3), visible desquamation (mean score 1.5 ± 0.1; 95% CI: 1.3–1.7), itching sensation (mean score 1.4 ± 0.1; 95% CI: 1.2–1.6), and skin tightness (mean score 1.7 ± 0.1; 95% CI: 1.5–1.9). At T28, 100% of participants achieved complete resolution of visible dryness and desquamation in the treated area according to the 5‐point scale (mean scores 0.0 ± 0.0; 95% CI: 0.0–0.0 for all parameters at T28) (all *p* < 0.001), though this may reflect a floor effect given mild baseline scores. No significant improvements were observed in the untreated leg.

#### 3.2.4 Self‐Reported Outcomes

Participant‐reported outcomes corroborated the instrumental and clinical findings. At T30min, 80.0% (95% CI: 66.8%–93.2%) reported reduced discomfort, while 88.6% (95% CI: 78.1%–99.1%) noted improvement in hydration. At T24h, 91.4% (95% CI: 82%.2–100.0%) reported overall improvement in skin appearance, together with increased hydration. At T28, 97.1% (95% CI: 91.6%–100.0%) agreed their skin felt more hydrated, 94.3% (95% CI: 86.7%–100.0%) expressed satisfaction with the product, 91.4% (95% CI: 82.2%–100.0%) stated they would recommend it, and 85.7% (95% CI: 74.1%–97.3%) indicated they would consider purchasing it.

#### 3.2.5 Safety and Tolerability

The test formulation was well tolerated by all participants. All safety monitoring procedures confirmed the absence of acute clinical irritation during the study period. However, the short duration of the study limits assessment of sensitization risk.

## 4 Discussion

Xerosis cutis is highly prevalent in clinical practice, and the associated signs and symptoms (scaling, desquamation, roughness, and pruritus) can be bothersome for patients and may negatively affect quality of life [18]. Findings from this randomized, intra‐individual controlled trial demonstrate that the tested topical formulation, comprising panthenol, niacinamide, and DPG, is effective in improving skin hydration and barrier function in subjects with xerosis cutis. A significant increase in corneometric hydration was observed as early as 30 min after the first application. However, such an early and marked increase is likely to reflect an immediate occlusive and humectant surface effect rather than structural barrier remodeling. Importantly, the persistence of improved hydration and the concomitant reduction in TEWL at later time points (T7 and T28) support a sustained functional benefit beyond the initial occlusive response. Although, TEWL reduction may reflect hydration‐mediated changes rather than structural lipid remodeling. The absolute reduction in TEWL at day 28 (from 9.2 to 7.9 g/h/m^2^; −1.3 g/h/m^2^) may appear modest in magnitude. However, in the context of mild‐to‐moderate xerosis, where baseline TEWL values are not severely elevated, even moderate reductions may reflect meaningful functional stabilization of the barrier. Importantly, TEWL improvement was consistent over time and paralleled sustained increases in corneometric hydration and clinical improvement, supporting a coherent barrier‐enhancing effect rather than an isolated instrumental fluctuation. Furthermore, the intra‐individual split‐leg design minimizes intersubject variability and allows direct comparison under identical systemic and environmental conditions.

Clinical assessments and participant‐reported outcomes corroborated these results: the convergence of instrumental data (epidermal hydration, TEWL), image‐based parameters (SEsc and SEr), and clinical/subjective outcomes reflects the formulation's significant value. Although SEr increased numerically by more than 50%, this represents an improvement in the definition of skin surface lines and overall. This should not be confused with clinical roughness, which is instead captured by the significant decline in the scaliness and desquamation parameters.

The efficacy of the topical formulation could be associated with the synergistic action of its active ingredients. Nonetheless, given the clinical trial design, this remains a hypothesis that requires further validation. Panthenol enhances hydration by binding water molecules and forming an occlusive film over the SC [19, 20]. It also promotes keratinocyte differentiation and re‐epithelialization [21]. In clinical settings, panthenol‐containing repair balms have shown favorable tolerance and improvements in barrier‐related outcomes in patients requiring regenerative care [12, 22], complementing consensus recommendations that basic emollients restore hydration and barrier function in xerosis [10]. Niacinamide acts at a cellular level to upregulate enzymes involved in the synthesis of key barrier lipids, thereby increasing ceramides, cholesterol, and free fatty acids [13, 23], and can improve stratum‐corneum biophysics and water‐handling properties [14, 24]. DPG exerts anti‐inflammatory effects by inhibiting pro‐inflammatory mediators implicated in pruritus such as IL‐8 and TNF‐α and suppressing NF‐κB activation [16]. Previous studies have evaluated the effects of panthenol and niacinamide individually or in simple combinations [13, 20]. However, to our knowledge, no head‐to‐head trials have evaluated this combination to date, limiting clinical positioning of this formulation. Future research should build upon these findings by incorporating vehicle‐controlled, double‐blind study designs to more precisely isolate the contribution of active ingredients. Expanding the study population to include ethnically diverse cohorts would enhance the generalizability of the results across different skin types. In addition, exploring molecular biomarkers related to lipid synthesis and cutaneous inflammation could provide further mechanistic insight into the formulation's mode of action. Longitudinal studies assessing long‐term efficacy, relapse rates, and durability of effects beyond the 28‐day application period are warranted. Finally, comparative trials with established ceramide‐based or prescription moisturizers would help position the formulation within current therapeutic frameworks and guide clinical decision‐making.

With respect to safety, all participants completed the study without discontinuation. Dermatologic examination revealed no signs of acute irritation and 100% of participants rated the product as well tolerated by day 28. This aspect is particularly relevant in individuals with an impaired skin barrier who are often prone to poor tolerance to certain formulations, especially those containing fragrances or superfluous additives.

## 5 Limitations

This study has several limitations. First, the lack of a vehicle control arm is a major methodological limitation that prevents us from attributing the observed effects solely to the active ingredients. The unblinded nature of the study for participants—inherent to the split‐leg design—may have introduced a significant response bias in the patient‐reported outcomes. Consequently, the high satisfaction and perceived efficacy rates should be interpreted with caution. Second, the study was conducted on a 100% Caucasian sample. This reduces the external validity of our findings, particularly for skin of color, where the clinical presentation and barrier dynamics of xerosis may present differently. Third, the cohort was 89% female. While this reflects common clinical attendance for xerosis, it may diminish the generalizability of the results to male populations. Fourth, the 28‐day duration of the trial precludes the assessment of relapse after treatment discontinuation or the durability of the formulation's effects across different seasons. Fifth, while the 5‐point ordinal scale (0–4) used for clinical assessment is standard in dermatological practice, it may lack the granular sensitivity required to distinguish between significant clinical improvement and absolute physiological resolution in a cohort with relatively mild baseline symptoms. This limitation, combined with a potential ‘floor effect’ given the mild baseline scores for dryness, may account for the observation that all participants reached a complete response (score of 0) by Day 28. Sixth, a specific correction for multiple comparisons was not applied despite the evaluation of multiple endpoints across six timepoints. This approach increases the risk of Type I error inflation, and the reported statistical significance should be interpreted with caution. Seventh, this study did not include histological analysis or lipid biomarker quantification. Therefore, the observed improvements reflect functional stabilization of the epidermal barrier rather than structural lipid matrix remodeling. Eighth, while participant compliance was monitored by weighing product containers at the end of the trial to verify total usage, this method does not provide real‐time data on. Consequently, while the total volume used was consistent with the study protocol, the exact frequency and timing of applications relied on participant self‐reporting. Finally, the device‐specific SELS parameters may not be directly translatable across different laboratory settings.

## 6 Conclusions

The topical formulation containing panthenol, niacinamide, and DPG appears to be an effective emollient with good tolerability for the management of xerosis. Instrumental, clinical, and subjective outcomes consistently supported its efficacy, and tolerability was excellent. However, within the context of a predominantly Caucasian and female cohort with mild‐to‐moderate baseline symptoms, these results likely reflect a functional stabilization of the epidermal barrier rather than definitive structural remodeling.

## Author Contributions

Daniel Morgado‐Carrasco led the study design, was involved in data collection and clinical assessments, and revised the manuscript writing. Pablo Balado‐Simó contributed to the interpretation of results and did the first writing of the manuscript. Fernando Vivancos Cuadras, Marta Furmanczyk, and Jesús Delgado Ojeda were responsible for statistical analysis and study design.

## Funding

The study was financially supported by Lacer. The funder provided financial support for the research but had no role in the data collection, interpretation of the results, or the writing of the manuscript. The final content of the manuscript was solely the responsibility of the cited authors.

## Disclosure

AI Disclosure: The authors confirm that no generative artificial intelligence or AI‐assisted technologies were used in the analysis, writing or preparation of this manuscript.

## Ethics Statement

The study was conducted in accordance with the Declaration of Helsinki, and approved by the Comitato Etico Indipendente per le Indagini Cliniche Non Farmacologiche on February 5th, 2025 (reference number 2025/03).

Informed consent was obtained from all subjects involved in the study.

## Conflicts of Interest

The authors declare no conflicts of interest.

## Supporting information


**Table S1:** Clinical assessment of the treated leg.

## Data Availability

The data that support the findings of this study are available from the corresponding author upon reasonable request.
