# Supplementary figures and images for: Efficacy of a Topical Formulation on Skin Hydration and Barrier Function in Individuals With Xerosis: A 28‐Day Randomized, Split‐Leg, Untreated‐Controlled Trial
Source: J Cosmet Dermatol. 2026 May 10;25:e70898. doi: 10.1111/jocd.70898 (PMC13158435; doi:10.1111/jocd.70898)

**Supplementary material**

**Table. Clinical assessment of the treated leg**

**
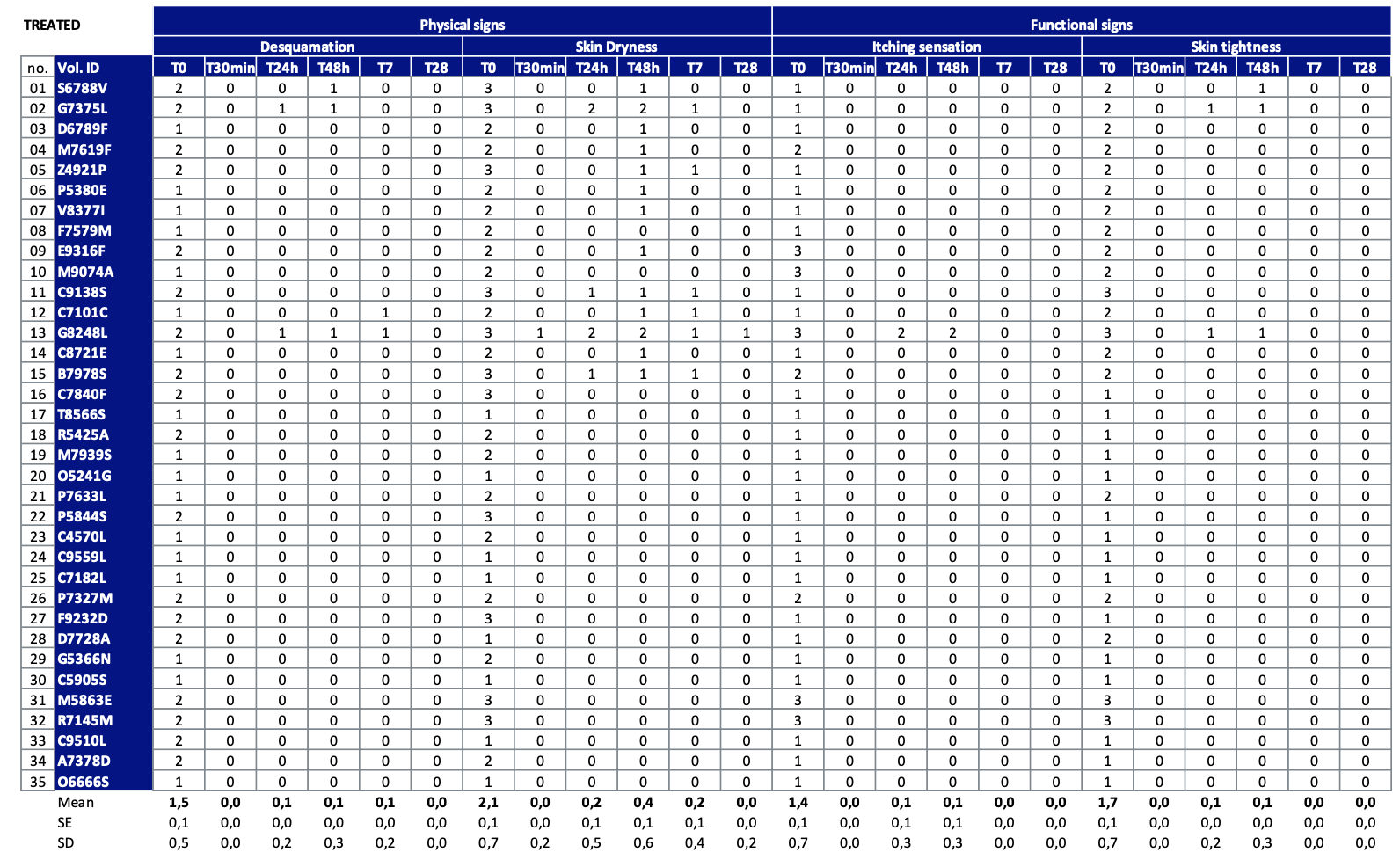
**

Supplement: Supplementary file 1 — Table S1: Clinical assessment of the treated leg. [file JOCD-25-e70898-s001.docx]
